# Comparing outcomes of systemic anticoagulant therapy and vascular interventional therapy in cerebral venous sinus thrombosis with concurrent brain parenchymal injury: Biomarker analysis

**DOI:** 10.5937/jomb0-55011

**Published:** 2025-08-21

**Authors:** Jinhui Qin, Xingyang Zhu, Xiaoli Wang, Xiangjie Cheng

**Affiliations:** 1 Nanyang Second General Hospital, Department of Cerebrovascular Intervention, Henan Province, China

**Keywords:** cerebral venous sinus thrombosis, brain parenchymal injury, anticoagulation, interventional thrombectomy, serum CCCK-18, neuron-specific enolase (NSE), S-100P, nerve growth factor (NGF) and CRP DDimer, tromboza cerebralnih venskih sinusa, oštećenje moždanog parenhima, antikoagulacija, intervencijska trombektomija, serumski CCCK-18, neuron-specifična enolaza (NSE), S-100P, faktor rasta nerva (NGF), CRP D-dimer

## Abstract

**Background:**

Cerebral venous sinus thrombosis (CVST) with concurrent brain parenchymal injury is a severe and complex condition that requires effective treatment strategies and long-term follow-ups. This study aimed to evaluate the prognostic value of serum caspase-cleaved cytokeratin-18 (CCCK-18), neuron-specific enolase (N SE), S-100P, nerve growth factor (N GF), and CRP D-Dimer, and EPO in CVST treatment.

**Methods:**

Ninety patients with CVST combined with parenchymal brain had undergone systemic anticoagulant therapy (SAT group) or vascular interventional therapy (VIT group) at Nanyang Second General Hospital from January 2021 to January 2024 were evaluated in this study with 45 patients in each group. Three months after discharge, mRS, NIHSS, GCS, and patients' quality of life were assessed. Peripheral blood samples were collected to measure CRP D-Dimer, EPO, and CCCK-18 level changes. The levels of serum neuron-specific enolase (NSE), S100P, and nerve growth factor (NGF) were compared before and at 3 and 7 days post-treatment. Follow-up at six months post-discharge included calculations of mortality and recanalisation rates.

**Results:**

At 3 months post-discharge, 11.1% of VIT patients had an mRSS2, compared to 35.6% in the SAT group (P< 0.05). The VIT group also had lower NIHSS scores, higher GCS and SF-36 scores, and lower serum CRP EPO, and CCCK-8 levels (P< 0.05). NSE and S-100P levels were lower in the VIT group at 7 days post-treatment (P< 0.05), while NGF levels were higher at 3 days post-treatment (P< 0.05). Follow-up showed no significant difference in survival rates (88.9% vs 95.6%). Still, the VIT group had a lower proportion of patients with mRS>2 (20.0% vs 42.2%) and a higher complete recanalisation rate (73.3% vs 53.3%) (both P< 0.05).

**Conclusions:**

Combined SAT with neurovascular interventional thrombectomy benefits patients with CVST and concurrent brain parenchymal injury by promoting recovery of neurological deficits and consciousness, achieving vascular recanalisation.

## Abstract (sr)

**Uvod:**

Tromboza cerebralnih venskih sinusa (CVST) sa istovremenim oštećenjem moždanog parenhima predstavlja ozbiljno i složeno stanje koje zahteva efikasne strategije lečenja i dugoročno praćenje. Cilj ove studije bio je da proceni prognostičku vrednost serumskih biomarkera, uključujući citokeratin-18 razgrađen kaspazom (CCCK-18), neuron-specifičnu enolazu (NSE), S-100P, faktor rasta nerva (NGF), kao i CRP D-dimer i EPO, u lečenju CVST-a.

**Metode:**

Devedeset pacijenata sa CVST-om u kombinaciji sa oštećenjem moždanog parenhima je podvrgnuto sistemskoj antikoagulantnoj terapiji (SAT grupa) ili vaskularnoj intervencijskoj terapiji (VIT grupa) u Drugoj opštoj bolnici u Nanyang-u od januara 2021. do januara 2024. godine, pri čemu je svaka grupa imala po 45 pacijenata. Tri meseca nakon otpusta ocenjivani su Mrs, NIHSS, GCS i kvalitet života pacijenata. Uzorci periferne krvi prikupljani su za merenje promena nivoa CRP-a, D-dimera, EPO-a i CCCK-18. Nivoi serumskih biomarkera neuron-specifične enolaze (NSE), S-100P i faktora rasta nerva (NGF) poređeni su pre tretmana, kao i trećeg i sedmog dana nakon tretmana. Praćenje šest meseci nakon otpusta uključivalo je procene stope mortaliteta i rekanalizacije.

**Rezultati:**

Tri meseca nakon otpusta, 11,1% pacijenata iz VIT grupe imalo je mRSS2, u poređenju sa 35,6% u SAT grupi (P< 0,05). Grupa VIT takođe je imala niže NIHSS skorove, više GCS i SF-36 skorove, kao i niže nivoe serumskog CRP-a, EPO-a i CCCK-18 (P< 0,05). Nivoi NSE i S-100P bili su niži u VIT grupi sedmog dana nakon tretmana (P< 0,05), dok su nivoi NGF-a bili viši trećeg dana nakon tretmana (P< 0,05). Praćenje nije pokazalo značajnu razliku u stopama preživljavanja (88,9% naspram 95,6%), ali je VIT grupa imala niži procenat pacijenata sa mRS>2 (20,0% naspram 42,2%) i višu stopu potpune rekanalizacije (73,3% naspram 53,3%) (oba P<0,05).

**Zaključak:**

Kombinacija sistemske antikoagulantne terapije (SAT) sa neurovaskularnom intervencijskom trombektomijom je od koristi pacijentima sa CVST-om i istovremenim oštećenjem moždanog parenhima, jer doprinosi oporavku neuroloških deficita i svesti, kao i postizanju vaskularne rekanalizacije.

## Introduction

Cerebral venous sinus thrombosis (CVST) is a relatively rare cerebrovascular disease, accounting for 0.5% to 1.0% of stroke patients, and is more common in young stroke populations [1]. The aetiology of CVST is complex, with diverse onset forms, and lacks specific clinical manifestations, resulting in relatively high rates of misdiagnosis, underdiagnosis, and mortality [2] [3]. Clinical manifestations of CVST are non-specific but are related to aetiology, lesion location, extent, progression rate, venous collateral circulation compensation, and secondary brain parenchymal injury range and severity. Clinical treatment modalities for CVST include anticoagulation, thrombolysis, and thrombectomy, with anticoagulation currently being the first-line treatment approach for CVST [4]. Anticoagulant therapy can effectively delay or inhibit further thrombus formation in CVST patients and prevent pulmonary embolism, but it cannot dissolve existing thrombi [5]. Thrombolytic therapy relies on thrombolytic agents encountering the thrombus to exert its therapeutic effect; however, its efficacy is limited for cortical vein thrombosis, deep vein thrombosis, and proximal catheter thrombosis [6].

Additionally, patients undergoing thrombolytic therapy are at risk of complications such as intracranial infection and vascular wall injury [7]. In clinical practice, vascular interventional thrombectomy is also used to treat CVST, especially for patients with concurrent brain parenchymal injury, as it achieves vascular recanalisation without the risks associated with thrombolysis [8] [9]. Vascular interventional thrombectomy can restore cerebral tissue perfusion and protect brain function, but it is an invasive procedure and carries potential risks. Inflammatory biomarkers have been found to play a significant role in CVST [10]. The utility of quantitative D-Dimer assay as a biomarker in diagnosing and excluding CVST has also been evaluated. D-Dimer assay is a sensitive biomarer for excluding deep vein thrombosis, and its utility in CVST has been extrapolated [11]. Other studies have also investigated the correlation between inflammatory biomarkers, the time evolution of CVST [12] and the prognostic value of the systemic immune-inflammation index in acute/subacute patients with CVST [13]. Research has shown that high serum levels of caspase-cleaved cytokeratin-18 are associated with increased mortality in patients with severe spontaneous intracerebral haemorrhage [14].

The quest for a reliable biomarker for acute ischemic stroke has led researchers to investigate the potential of neuron-specific enolase (NSE) as a predictor of brain damage caused by infarction [15]. NSE, an enzyme released after neuronal damage, has been studied extensively as a marker for brain injury, including cerebral infarction [16]. The temporal profile and clinical significance of serum NSE have been explored in various studies, with some indicating a correlation between NSE levels and infarct volume, as well as neurological worsening in cerebrovascular stroke [17]. Furthermore, research has shown that NSE levels at admission can serve as a valuable marker to predict stroke severity and early functional outcomes [18]. The intricate relationship between nerve growth factor (NGF) and brain damage has been the subject of extensive research, with studies [19] [20] [21] [22] [23] elucidating the role of NGF in promoting neural recovery and plasticity after brain injury. NGF has been shown to support neuronal growth, differentiation, and survival of brain cells, thereby mitigating the effects of traumatic brain injury [20] [23]. The administration of NGF has been found to increase the activity of antioxidant enzymes in brain tissues, reduce the overload of calcium ions, and provide protective effects on neurons after traumatic brain injury [22]. Furthermore, NGF has been demonstrated to have therapeutic potential in neurodegenerative diseases, with its ability to modulate immune function and attenuate cognitive deficits [19] [21]. The intranasal administration of NGF has been shown to improve neurological outcomes in patients with severe traumatic brain injury and meningitis [20] [23].

As the current treatment strategies for CVST with concurrent brain parenchymal injury are often hindered by limitations and risks associated with anticoagulation, thrombolysis, and thrombectomy, we aimed to evaluate the prognostic value of various biomarkers and compare the outcomes of systemic anticoagulant therapy (SAT) and vascular interventional therapy (VIT) in patients with CVST The existing literature is marred by a lack of comprehensive studies investigating these treatment modalities' long-term effects, particularly in biomarker analysis and patient outcomes. Furthermore, the optimal treatment approach for CVST with concurrent brain parenchymal injury remains unclear, highlighting the need for a comparative study that assesses the efficacy and safety of SAT and VIT. This study is novel in that it not only compares the outcomes of SAT and VIT but also explores the prognostic value of serum biomarkers, including caspase-cleaved cytokeratin-18, neuron-specific enolase, S-100β, nerve growth factor, CRP D-Dimer, and EPO, in patients with CVST, providing valuable insights into the pathophysiology and treatment of this complex condition.

## Materials and methods

### Case information

A total of 90 patients diagnosed with CVST combined with brain parenchymal injury at Nanyang Second General Hospital from January 2021 to January 2024 were recruited. Diagnostic criteria for CVST: presence of symptoms such as headache, vomiting, indicative of increased intracranial pressure, and/or other neurological manifestations; digital subtraction angiography (DSA) showing incomplete or complete non-visualisation of intracranial venous sinuses with or without venous collateral circulation; for patients who did not undergo DSA, magnetic resonance imaging (MRI) or MR venogram (MRV) showing disappearance of intracranial venous sinus blood flow and thrombus signals at different periods. The patients were then randomly divided into the systemic anticoagulant therapy group (SAT group) and the vascular interventional therapy group (VIT group), with 45 patients in each group. This experiment obtained approval from the Medical Ethics Committee of Nanyang Second General Hospital.

Inclusion criteria: diagnosis of CVST confirmed by imaging and laboratory tests; complete clinical data available for the case; evidence of brain parenchymal injury such as cerebral contusion, intracranial hematoma, or cerebral infarction shown on cranial MRI or other imaging studies; accompanied by a family member who understands the study content and signs the informed consent form. Exclusion criteria: the presence of other intracranial diseases such as arteriovenous fistula or malformation; coexisting conditions like pulmonary embolism, deep vein thrombosis in the lower extremities, or other thrombotic disorders; history of prior intracranial surgery before treatment; significant dysfunction of vital organs such as heart, liver, or kidneys; malignant tumours.

### Therapeutic methodologies

After the aetiology was determined, all patients received active treatment for their underlying conditions. Patients with infections were administered antimicrobial therapy, those with severe dehydration received electrolyte balance maintenance therapy, patients with elevated blood viscosity were treated with volume expansion, those experiencing seizures were managed with antiepileptic therapy, and patients with intracranial hypertension underwent intracranial pressure-lowering treatments. Additionally, medications were administered to nourish brain cells and improve cerebral circulation [24].

Patients in the SAT group received SAT based on basic and symptomatic treatment. This involved subcutaneous injections of low molecular weight heparin (approval number: National Drug Approval H19990035; Manufacturer: Hangzhou Jiuyuan Gene Engineering Co., Ltd.; Specification: 0.3 mL: 3,000 IU) twice daily (180 anti-Xa factor activity units per dose). Before discharge, patients were transitioned to oral warfarin (approval number: National Drug Approval H20054247; Manufacturer: Beijing Julin Pharmaceutical Co., Ltd.; Specification: 5 mg) for anticoagulation therapy. Regular monitoring of coagulation tests was performed, and warfarin dosages were adjusted promptly (targeting prothrombin time of 25~30 s and international normalised ratio of 2~3).

Patients in the VIT group received intravenous sinus intervention thrombectomy in addition to basic, symptomatic, and SAT. The procedure involved a modified Seldinger technique with a right femoral artery puncture and placement of a 5F sheath for diagnostic cerebral angiography. Thrombus extent and severity were determined based on criteria including cerebral circulation time, cerebral venous sinus appearance, and jugular vein engorgement. Cerebral circulation time >9 s was considered abnormal. Next, a 6F sheath was placed via the right femoral vein puncture to guide a catheter into the proximal internal jugular vein at the base of the skull where thrombosis originated, and continuous heparinisation was administered. A stent retrieval device was manoeuvred into position using venous access, with the microcatheter advanced to the thrombus distal end after withdrawal of the micro guidewire. The Solitaire stent was deployed at the thrombus site and subsequently retracted along with the micro guidewire, with periodic blood aspiration to prevent pulmonary embolism. Thrombectomy was repeated 3-5 times as necessary until the primary venous sinus was patent. Subsequent DSA confirmed venous sinus flow restoration, and brain parenchymal lesions were assessed for worsening or new developments. Postoperative anticoagulation therapy was initiated with the same protocol as the SAT group.

### Clinical efficacy evaluation

At 3 months post-discharge, 3 mL of venous blood was collected from patients to examine serum C-reactive protein (CRP), D-dimer (D-D), erythropoietin (EPO), and CCCK-18). Furthermore, fasting venous blood samples were collected from patients before and at 3 days and 7 days post-treatment, with each sample volume being 3 mL. After centrifugation at 3,000 rpm for 10 minutes, serum was collected. Serum levels of neuron-specific enolase (NSE), S-100β, and nerve growth factor (NGF) were measured using the Siemens ADVIA 1800 fully automated biochemical analyser.

The evaluation of patients' independence in daily living at three months post-discharge was performed using the modified Rankin Scale (mRS), which assesses physical function, activity ability, and activities of daily living. For scores, 0 indicates no symptoms; 1 indicates no significant symptoms or signs; 2 indicates slight symptoms not affecting daily life; 3 indicates mild limb disability affecting daily life but capable of independent walking; 4 indicates moderate limb disability requiring assistance with daily activities; and 5 indicates severe limb disability, bedridden, and requiring constant care. A prognosis of »good« is defined as mRS 2, while mRS>2 or death indicates a »poor« prognosis.

The evaluation of patients' neurological deficits at 3 months post-discharge was conducted using the National Institutes of Health Stroke Scale (NIHSS), which assesses consciousness level, vision, facial muscle movement, limb motor function, balance and coordination, sensory function, language and comprehension abilities, speech expression, attention, and concentration. The total score is 42 points, with higher scores implying more severe neurological deficits.

The assessment of patients' level of consciousness at 3 months post-discharge was performed using the Glasgow Coma Scale (GCS), which assesses eye response, verbal response, and motor response. The total score is 15 points, with higher scores indicating better consciousness status.

The patient's quality of life at 3 months post-discharge was evaluated using the 36-Item Short Form Survey (SF-36), which evaluates physical function, physical role, physical pain, general health, vitality, social function, emotional role, and psychological health. The total score is 100 points, with higher scores suggesting a better quality of life.

Follow-up was conducted for at least six months post-discharge to record adverse events and mortality occurrences among patients, with prognosis assessed using the mRS.

### Data processing and analysis

The case and indicator data were organised using Excel, and statistical analysis was implemented using SPSS 23.0. Binary categorical variables were presented as n (%) and compared employing χ^2^ test. Continuous variables were denoted as mean ± SD and compared employing a t-test. Using the Log-rank test, Kaplan-Meier curves were plotted to compare survival rates between the two groups. *P*<0.05 meant a statistically significant difference.

## Results

### Comparison of general patient information

The differences in general characteristics between patients in the SAT and VIT groups were analysed and compared (Table 1). It was found that neglectable differences (*P*>0.05) existed between groups regarding age, gender, onset characteristics, onset triggers (including infectious causes such as pulmonary infections, sepsis, and sinusitis; and noninfectious causes such as oral contraceptive use history, trauma, nephrotic syndrome, blood component abnormalities, pregnancy/postpartum period), and distribution of brain parenchymal injuries.

**Table 1:** Comparison of general information.

Data	SAT group (*n*=45)	VIT group (*n*=45)	*P*
Age, years old	33.5±4.1	35.1±4.4	0.433
Sex, *n* (%)			0.210
Male	29 (64.4)	27 (60.0)	
Female	16 (35.6)	18 (40.0)	
Characteristics of onset, *n *(%)			0.365
Acute	16 (35.6)	15 (33.3)	
Subacute	20 (44.4)	22 (48.9)	
Chronic	9 (20.0)	8 (17.8)	
Causes of onset, *n* (%)			0.104
Infectious etiology	4 (8.9)	5 (11.1)	
Non-infectious etiology	33 (73.3)	30 (66.7)	
No obvious cause was found	8 (17.8)	10 (22.2)	
Distribution of area of parenchymal<br>brain injury, *n* (%)			0.297
Parietal lobe	16 (35.6)	15 (33.3)	
Frontal lobe	11 (24.4)	13 (28.9)	
Temporal lobe	9 (20.0)	10 (22.2)	
Occipital lobe	9 (20.0)	6 (13.3)	
Thalamus basal ganglia	8 (17.8)	6 (13.3)	
Brainstem	3 (6.7)	2 (4.4)	
Cerebellum	2 (4.4)	2 (4.4)	

### Comparison of clinical characteristics of patients

**Figure 1:**
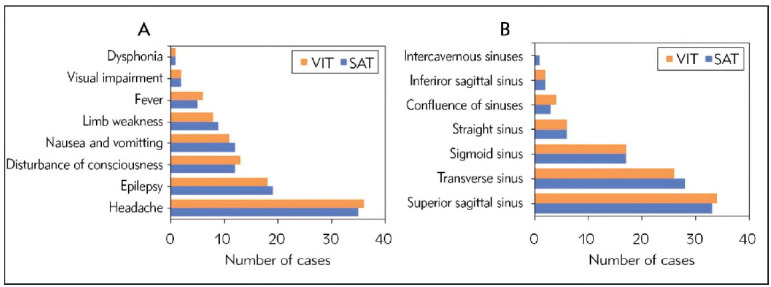
Comparison of clinical manifestations between two groups (A represents clinical features, В represents imaging features).

First, the differences in clinical manifestations between patients in the SAT and VIT groups were analysed and compared (Figure 1A). It was found that headache was the primary symptom in all patients, followed by epilepsy and then disturbance of consciousness. In the SAT group, headache accounted for 77.8% (35 cases), epilepsy accounted for 42.2% (19 cases), disturbance of consciousness accounted for 26.7% (12 cases), nausea and vomiting accounted for 26.7% (12 cases), limb weakness accounted for 20.0% (9 cases), fever accounted for 11.1% (5 cases), visual impairment accounted for 4.4% (2 cases), and dysphonia accounted for 2.2% (1 case). Similarly, in the VIT group, headache accounted for 80.0% (36 cases), epilepsy accounted for 40.0% (18 cases), disturbance of consciousness accounted for 28.9% (13 cases), nausea and vomiting accounted for 24.4% (11 cases), limb weakness accounted for 17.8% (8 cases), fever accounted for 13.3% (6 cases), visual impairment accounted for 4.4% (2 cases), and dysphonia accounted for 2.2% (1 case). The differences in clinical manifestations between the two groups were inconsiderable (*P*>0.05). Next, the differences in imaging characteristics between patients in the SAT and VIT groups were compared (Figure 1B). It was found that lesions involving the superior sagittal sinus were relatively common, with the SAT and VIT groups showing lesions in the superior sagittal sinus accounting for 73.3% (33 cases) and 75.6% (34 cases), respectively. Similarly, lesions involving the transverse sinus were observed in 62.2% (28 cases) of the SAT group and 57.8% (26 cases) of the VIT group. In comparison, lesions involving the sigmoid sinus were present in 37.8% (17 cases) of both the SAT and VIT groups. Additionally, involvement of the straight sinus was observed in 13.3% (6 cases) of the SAT group and 13.3% (6 cases) of the VIT group, with the confluence of sinuses affected in 6.7% (3 cases) of the SAT group and 8.9% (4 cases) of the VIT group. Furthermore, lesions in the inferior sagittal sinus were found in 4.4% (2 cases) of both the SAT and VIT groups, while the involvement of the intercavernous sinuses was observed in 2.2% (1 case) of the SAT group and 0.0% (0 cases) of the VIT group. The differences in imaging characteristics between the two groups were insignificant (*P*>0.05).

### Comparison of clinical efficacy among patients

**Figure 2:**
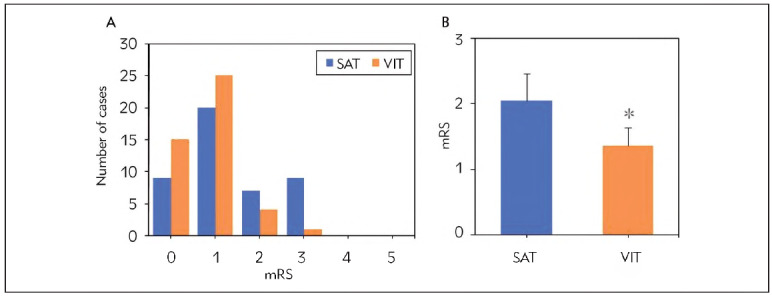
Comparison of mRS scores between two groups (A represents clinical features, B represents imaging features; *P<0.05 vs. SAT group).

First, the differences in mRS scores between patients in the SAT and VIT groups at discharge three months post-admission were analysed and compared (Figure 2). It was found that in the SAT group, the distribution of mRS scores was as follows: 0 points, 9 cases (20.0%); 1 point, 9 cases (20.0%); 2 points, 7 cases (15.6%); 3 points, 9 cases (20.0%); 4 points and 5 points, 0 cases (0.0%). Among them, 16 cases (35.6%) had mRS scores ≥2, and 34 cases (64.4%) had mRS scores <2. In contrast, in the VIT group, the distribution of mRS scores was as follows: 0 points, 15 cases (33.3%); 1 point, 25 cases (55.6%); 2 points, 4 cases (8.9%); 3 points, 1 case (2.2%); 4 points and 5 points, 0 cases (0.0%). Among them, 5 cases (11.1%) had mRS scores ≥2, and 40 cases (88.9%) had mRS scores <2. The proportion of mRS scores ≥2 and the mean mRS score were markedly lower in the VIT group versus the SAT group (*P*<0.05).

Next, the differences in NIHSS, GCS, and SF-36 scores between patients in the SAT and VIT groups at discharge three months post-admission were analysed and compared (Table 2). It was found that the NIHSS scores were lower in the VIT group versus the SAT group, while the GCS scores and SF-36 scores were higher in the VIT group versus the SAT group (*P*<0.05).

**Table 2:** Comparison of NIHSS, GCS, and SF-36 scores between two groups (mean±SD).

Item	SAT group<br>(*n*=45)	VIT group<br>(*n*=45)	*P*
NHISS	3.4±0.3	1.9±0.3	0.000
GCS	12.3±1.1	14.2±1.2	0.000
SF-36	83.5±5.4	89.1±6.8	0.000

### Comparison of patient serological indicators

The differences in serum levels of CRP D-D, EPO, and CCCK-18 between patients in the SAT and VIT groups at discharge three months post-admission were analysed and compared (Table 3). CRP EPO, and CCCK-18 serum levels were lower in the VIT group versus the SAT group (*P*<0.05).

**Table 3:** Comparison of serological indexes between the two groups (mean±SD).

Index	SAT group<br>(*n*=45)	VIT group<br>(*n*=45)	*P*
CRP (mg/L)	13.63±1.24	9.38±1.52	0.000
D-D (μg/mL)	1.12±0.11	1.34±0.14	0.000
EPO (mU/mL)	107.21±7.69	86.57±8.41	0.000
CCCK-8 (U/L)	183.25±13.32	144.96±15.43	0.000

**Figure 3:**
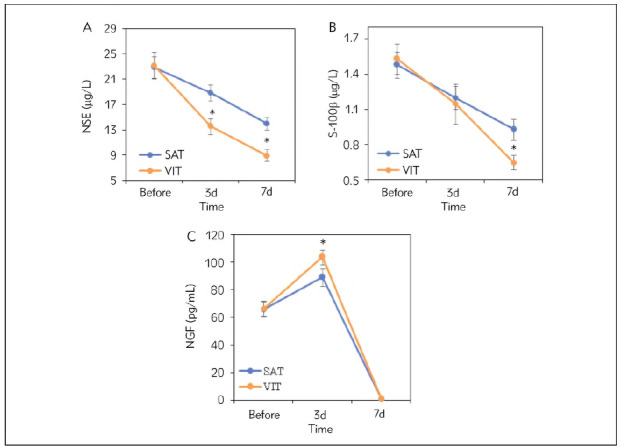
Serum levels of NSE, S-100β, and NGF at different time points (A represents NSE, and B represents S-100β, while C represents NGF).

The analysis compared the serum levels of NSE, S-100β, and NGF in patients of the SAT and VIT groups before treatment, at 3 days post-treatment, and 7 days post-treatment (Figure 3). Before treatment, there were no statistically significant differences in NSE, S-100β, and NGF levels between the VIT and SAT groups (*P*>0.05). After treatment, the VIT and SAT groups showed a decreasing trend in NSE and S-100β levels. The levels of NSE at 3 days and 7 days post-treatment were significantly lower in the VIT group compared to the SAT group (*P*<0.05), and the S-100β levels at 7 days post-treatment were lower in the VIT group than in the SAT group (*P*<0.05). Post-treatment, both the VIT and SAT groups exhibited a trend of initially increasing and then decreasing NGF levels. At 3 days post-treatment, the NGF levels were higher in the VIT group compared to the SAT group (*P*<0.05), while at 7 days post-treatment, there were no statistically significant differences in NGF levels between the two groups (*P*>0.05).

### Comparison of prognosis of patients

**Figure 4:**
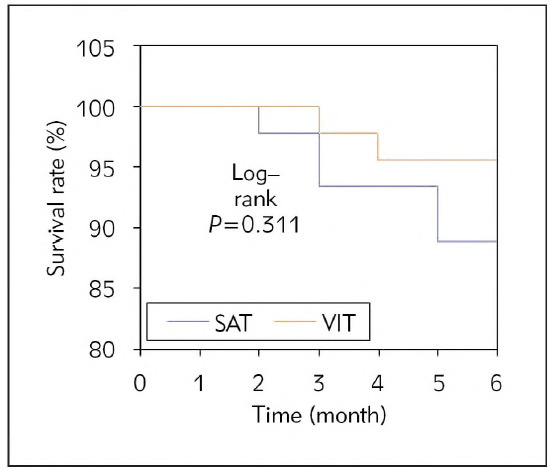
Two groups of Kaplan-Meier curves

The analysis compared the differences in prognosis between groups at 6 months of follow-up (Figure 4). First, the survival outcomes of both groups were analysed (Figure 3). In the SAT group, there were 5 deaths, demonstrating a survival rate of 88.9%, while in the VIT group, there were 2 deaths, demonstrating a survival rate of 95.6%. However, this difference was neglectable (*P*>0.05).

**Figure 5:**
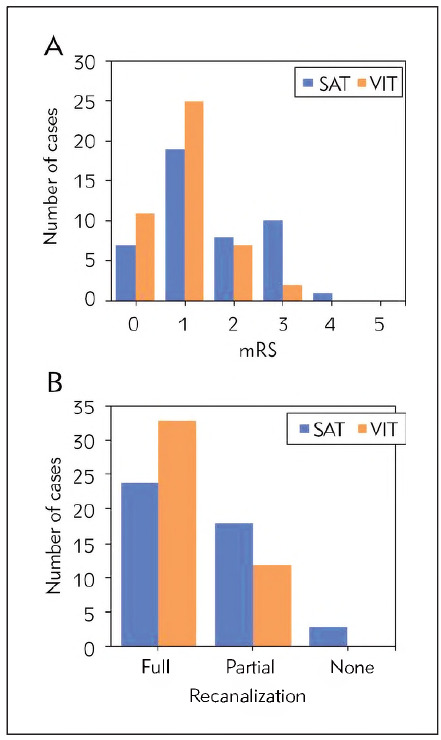
Comparison of prognosis between the two groups (A is mRS score, and B is vascular recanalisation).

Next, the mRS scores of the two groups were analysed (Figure 5A). In the SAT group, the distribution of mRS scores was as follows: 0 points, 7 cases (15.6%); 1 point, 19 cases (42.2%); 2 points, 8 cases (17.8%); 3 points, 10 cases (22.2%); 4 points, 1 case (2.2%); 5 points, 0 cases (0.0%). Among them, 19 cases (42.2%) had mRS scores ≥2, and 26 cases (57.8%) had mRS scores <2. In the VIT group, the distribution of mRS scores was as follows: 0 points, 11 cases (24.4%); 1 point, 25 cases (55.6%); 2 points, 7 cases (15.6%); 3 points, 2 cases (4.4%); 4 points and 5 points, 0 cases (0.0%). Among them, 9 cases (20.0%) had mRS scores ≥2, and 36 cases (80.0%) had mRS scores <2. The proportion of mRS scores ≥2 in the VIT group was drastically inferior to that in the SAT group (*P*<0.05). Finally, the recanalisation rates of the two groups were compared (Figure 5B). In the SAT group, 24 cases (53.3%) achieved complete recanalisation, 18 cases (40.0%) achieved partial recanalisation, and 3 cases (6.7%) showed no change. In the VIT group, 33 cases (73.3%) achieved complete recanalisation, 12 cases (26.7%) achieved partial recanalisation, and 0 cases (0.0%) showed no change. The recanalisation rate in the VIT group was markedly superior to the SAT group (*P*<0.05).

## Discussion

Our study investigated the value of serum CCCK-18, neuron-specific enolase (NSE), S-100β, nerve growth factor (NGF), and CRP D-Dimer, and EPO after CVST treatment and compared the outcomes of systemic anticoagulant therapy and vascular interventional therapy in patients with CVST and concurrent brain parenchymal injury. In contrast, previous studies [25] [26] [27] [28] have focused on the clinical differences between acute CVST and non-thrombotic CVSS, the association between CVST and high altitude, and biomarkers such as D-dimer and fibrinogen in predicting acute CVST. Our study found that the vascular interventional therapy group had better outcomes, including lower mRS scores, higher GCS scores, and lower serum CRP EPO, and CCCK-18 levels than the systemic anticoagulant therapy group. This is consistent with the findings of other studies [25] [26] [27] [28] highlighting the importance of early and effective treatment in improving outcomes in CVST patients. However, our study is unique in focusing on the prognostic value of specific biomarkers and comparing different treatment strategies in patients with CVST and concurrent brain parenchymal injury.

Mohammadian et al. [29] demonstrated that endovascular thrombolysis for progressive CVST is safe and effective. This study found that compared to sole SAT, patients treated with combined endovascular thrombectomy had a lower proportion of mRS scores ≥2, lower NIHSS scores, and higher GCS and SF-36 scores. Endovascular thrombectomy rapidly restores venous sinus patency, blood circulation, and improves patients' intracranial hypertension and cerebral blood flow. Therefore, combining anticoagulation therapy with endovascular thrombectomy maximises the restoration of neurological function in CVST patients and is beneficial for prognosis and outcomes [30].

Vascular pathology forms the basis of CVST, and inflammatory factors also play a crucial role in this pathology. CRP is an acute-phase reactant protein that can cause vascular and endothelial damage [31] [32] [33]. Moreover, CRP, as an inflammatory marker, is closely associated with thrombus formation, a chronic inflammatory process involving vascular formation. This study found that compared to sole SAT, patients treated with combined thrombectomy showed reduced serum CRP levels. This suggests that combined SAT with thrombectomy can lower serum CRP levels in patients with CVST and brain parenchymal injury, potentially reducing postoperative thrombus formation and favouring vascular patency. D-D may be an effective indicator for diagnosing and treating suspected CVST patients [34]. Hypercoagulability and thrombolytic therapy can induce elevated D-D levels, which may increase with disease severity [35]. Thrombus degradation can lead to increased D-D levels in the blood. This study found a neglectable difference in serum D-D between patients treated with sole SAT and those treated with combined thrombectomy, indicating that both methods effectively contribute to thrombus clearance. EPO is a glycoprotein produced by the kidneys that primarily promotes hematopoiesis and can be used as an adjunct diagnostic tool for conditions such as renal anaemia, aplastic anaemia, and myelodysplastic syndromes [36]. A high red blood cell count increases blood viscosity, making it more prone to thrombus formation within blood vessels, thereby elevating the risk of thrombosis [37]. Cytokeratin (CK) primarily exists in brain microvascular endothelial cells, glial cells, and meningeal cells. It is released into the bloodstream following cellular apoptosis or breakdown [38]. CCCK-18 serves as a potential biological indicator for assessing prognosis in neurological diseases, participating in the acute onset of such conditions. This study found that patients treated with combined thrombectomy exhibited significant reductions in serum levels of EPO and CCCK-18 relative to sole SAT. Higher serum CCCK-18 levels correlate with more severe degrees of brain parenchymal cell apoptosis and, consequently, more severe secondary brain damage [14]. Lastly, this study's follow-up revealed that compared to sole SAT, patients treated with combined thrombectomy had a lower proportion of follow-up mRS scores ≥2 and higher vascular patency rates. Sole SAT combined with thrombectomy can mitigate further exacerbation of brain injury, hinder thrombus formation, and improve patient prognosis by reducing serum levels of EPO and CCCK-18 in patients with CVST and brain parenchymal injury.

NSE is an acidic protein enzyme widely distributed in brain neurons and endocrine cells. It is released when brain tissue is damaged or cell membranes are disrupted, thus serving as a specific marker for neuronal damage. Studies suggested a positive correlation between serum NSE levels and the number of neuronal cell deaths in the body [39]. This study indicated that after treatment, the NSE levels in both groups of patients showed a decreasing trend, possibly reflecting the treatment's impact on neuronal damage. Particularly on the 3rd and 7th days posttreatment, the NSE levels in the VIT group were lower than those in the SAT group, suggesting that vascular intervention therapy may be more effective in reducing neuronal damage compared to systemic anticoagulant therapy. S-100β is an inhibitory soluble acidic calcium-binding protein present in nervous tissue, with low levels in serum and cerebrospinal fluid in non-neurological disorders. However, soluble S-100β enters the cerebrospinal fluid once neurons, small glial cells, or astrocytes are damaged, then crosses the blood-brain barrier into the bloodstream.

Furthermore, S-100β can increase its synthesis and secretion through acute glial reactions, participating in the repair process of brain injury. Therefore, serum S-100β levels can reflect the extent of damage and death of central nervous cells [40]. The results indicated that the levels of S-100β in both groups of patients showed a decreasing trend after treatment, suggesting that both modalities were beneficial for central nervous cell damage. Particularly, on the 7th day post-treatment, the S-100β levels in the VIT group were lower than those in the SAT group, indicating that vascular intervention therapy had a better effect in reducing the extent of neuronal damage. NGF belongs to the family of neurotrophic factors, regulating the intake of amino acids and synthesising functional proteins, thereby promoting neuronal regeneration and injury repair to a certain extent, participating in the protective process of brain ischemic injury [41].

Additionally, NGF also protects neurons, alleviating brain ischemia-hypoxia and toxic damage. The results in the text show that the NGF levels in both groups of patients exhibited a trend of initial increase followed by a decrease after treatment, reflecting the dynamic changes of neurotrophic factors after treatment. NGF may play an essential role in promoting neuronal regeneration and injury repair, thus showing a trend of NGF levels changing with treatment time. On the 3rd day post-treatment, the NGF levels in the VIT group were higher than those in the SAT group, while on the 7th day post-treatment, the NGF levels in the VIT group were lower than those in the SAT group. These results indicate that at different time points, the two treatment modalities may have different effects on NGF levels, suggesting that different treatment modalities may affect the expression levels of neurotrophic factors, thereby influencing the processes of neuronal regeneration and injury repair. However, further research is needed to elucidate these differential effects' mechanisms and clinical significance. Overall, these results suggest that in treating CVST patients with concomitant cerebral parenchymal injury, vascular intervention therapy (VIT) may be more effective in reducing neuronal damage and central nervous cell injury than systemic anticoagulant therapy (SAT).

This study also has certain limitations. In the future, it is necessary to include more samples and data to analyse further the risk factors affecting the prognosis of CVST combined with parenchymal brain damage.

## Conclusion

This study aimed to evaluate the long-term outcomes and biomarker changes in patients with CVST and concurrent brain parenchymal injury treated with systemic anticoagulant therapy (SAT group) or vascular interventional therapy (VIT group). The results indicate that the VIT group had better clinical outcomes and favourable biomarker profiles than the SAT group. Specifically, at 3 months post-discharge, 11.1% of VIT patients had an mRS ≥2, compared to 35.6% in the SAT group (P<0.05). The VIT group also had lower NIHSS scores, higher GCS, better SF-36 scores, and lower serum CRP EPO, and CCCK-8 levels (P<0.05). NSE and S-100β levels were significantly lower in the VIT group at 7 days post-treatment (P<0.05), while NGF levels were higher at 3 days post-treatment (P<0.05).

## Dodatak

### Conflict of interest statement

All the authors declare that they have no conflict of interest in this work.
